# The Effect of Maternity Support Garments on Alleviation of Pains and Discomforts during Pregnancy: A Systematic Review

**DOI:** 10.1155/2019/2163790

**Published:** 2019-08-01

**Authors:** Carolina Quintero Rodriguez, Olga Troynikov

**Affiliations:** ^1^Human Ecology and Clothing Science Research Group, RMIT University, 3056, Australia; ^2^School of Engineering, College of Science, Engineering and Health, RMIT University, 3083, Australia

## Abstract

Maternity support garments (MSGs) are widely available and commonly recommended and used for alleviation of lower back pain (LBP) and pelvic girdle pain (PGP) during pregnancy; however, most studies available use the garments as a conjunct intervention with other therapies, with scarce research demonstrating the effects of the garments as a sole intervention. This study aims to review the available literature on the effects of using MSGs as sole intervention for comfort improvement of women during pregnancy, as well as to discuss the attributes of the garments which may influence their performance. A systematic review was undertaken, which adheres to PRISMA guideline for systematic reviews. Multiple databases, such as ScienceDirect, CINAHL, EBSCO, Elsevier, SCOPUS, Wiley Online Library, ProQuest, ProQuest Health and Medical Complete, PubMed, and Cochrane Central Register of Controlled Trials, were electronically searched. Six studies met the inclusion criteria and covered three trial studies, two pilot studies and one observational study. Three outcome measurements were identified from the included studies: alleviation of pain, improvement of balance, and improvement of functionality and mobility. The study concluded that wearing MSGs during pregnancy could have beneficial effects in women such as LBP and PGP alleviation, improvement of functionality and mobility, and reduction of risk of fall during pregnancy; however, the mechanisms of the garments' actions as well as the duration of the garments' effectiveness are not elucidated through the studies. This study contributes to the understanding of the effects and effectiveness of the use of MSGs as a sole intervention for improvement of comfort during pregnancy as well as information about the different types of garments commercially available and the attributes that may influence the garment performance.

## 1. Introduction

Musculoskeletal discomforts and pains are common during pregnancy. 20% of women experience pelvic girdle pain (PGP) and more than 65% of women experience lower back pain (LBP), with the pains occurring separately or concurrently and interfering with the performance of Activities of Daily Living (ADL), compromising women's quality of life (QOL), and in some cases leading to absenteeism and even disability [1–3]. Mogren and Pohjanen [4] added that the relapse rates related to LBP and PGP observed in subsequent pregnancies are high, with prevalence of 24.7% during postpartum.

There are many hypothesized aetiologies of pregnancy-related LBP and PGP including mechanical/anatomical changes and hormonal changes that affect the sacroiliac joint (SIJ) and the pubis symphysis on the pelvic bone resulting in joint laxity as well as inflammatory, vascular and neural (peripheral and central) factors [5, 6].

The European guidelines for the diagnosis and treatment of PGP [7] and prenatal practitioners in the United Kingdom and Nordic countries recommend that LBP and PGP treatments include information for patients to be active and to continue normal daily activities, offering specific individual exercises where appropriate and/or referring these patients to physiotherapy for more specific treatment. Other nonpharmacologic treatments recommended include exercise, rest, massage, acupuncture, hot and cold compress, chiropractic, relaxation, aromatherapy, yoga, Reiki and the use of maternity support garments (MSGs) [8, 9].

MSGs are garments designed with the purpose of supporting the abdomen and lower back of pregnant women, caring for the safety of the developing baby [10]. These garments have been denominated as convenient, safe, low-cost and easily accessible therapy to manage LBP and PGP and are frequently recommended by specialists and worn by pregnant women [11, 12].

Ho, Luo [13] mentioned four main types of commercially available garments in the form of panties or briefs, belts or girdles, cradle, and torso supports which intend to decrease pregnancy-related pain by offering support in the lumbar spine and/or pelvic regions [14]. Some of the hypothesised effects of the garments are related to the provision of mechanisms to compress the body, increasing body proprioception, limiting spinal motion, stabilizing the lumbar spine and/or pelvis, reducing mechanical loading of localized weight, stimulating the action of the muscles around the abdomen, the spine, and the pelvic floor, and/or offering a placebo effect to the wearer [2, 15]. However, it is common for specialists to recommend the garments as a conjunct therapy to other therapies, which makes it difficult to elucidate its effectiveness as a sole intervention.

The aim of this study is to review the available research on the effects of using MSGs as a sole intervention during pregnancy and to analyse the state of the art in MSGs on design principles, features, and materials and constructions, which may influence the garments' performance.

## 2. Methods

The term “support garment” in the present review is used most broadly and all types of garments that aim to alleviate pregnancy-related discomforts or pains such as LBP, PGP, symphyseal pain, and lumbopelvic pain were considered.

Firstly, a systematic review of patented designs and commercially available MSGs took place from August to October 2017. Databases such as Espacenet and USPTO were used for patents search and electronic engine Google was used for search of commercially available garments. Visits to pharmacies and orthopedic retail stores where feasible were also undertaken.

Secondly, an extensive literature search was conducted using relevant electronic databases: ScienceDirect, CINAHL, EBSCO, Elsevier, SCOPUS, Wiley Online Library, ProQuest, ProQuest Health and Medical Complete, PubMed, and Cochrane Central Register of Controlled Trials. In addition, a search was conducted using Google Scholar. The literature search strategy combined the use of three primary keywords: “support garments”, pregnan*∗*, and belt*∗*, combined with other keywords like matern*∗*, LBP, PGP, lumbopelvic pain, symphysis pubis dysfunction, symphyseal pain, back pain, pelvic pain, sacroiliac joint laxity, vest, brief, bands, cradle, pain, discomfort, pressure garment, and compression garment.

The literature search was performed from October 2017 to August 2018, where the databases were accessed multiple times and relevant studies were examined for inclusion in this review. Relevant papers were identified through their titles and abstracts. After the publications were retrieved, the first author reviewed them to determine the suitability.

The inclusion selection criteria included studies in English, carried out with pregnant women of any gestational age, experiencing any pregnancy-related discomfort or pain, and using any type of garment as a main treatment for its alleviation. Studies were included if the garment was used as a stand-alone intervention or if the use of the garment was accompanied by information provided to a patient with respect to the body anatomy, posture, and physiology, the reasons for the emergence of discomforts, benefits of maintaining activity during pregnancy, and others.

The exclusion criteria covered systematic review papers and studies that investigated the use of MSGs as an adjunct treatment to other interventions such as manual therapies, acupuncture, exercise, and others, where separate effect of MSGs was not clearly determined on the outcome(s). For example, the clinical trial by Depledge, McNair [16], which investigated the combined effects of exercise, advice, and pelvic support belts on the management of symphysis pubis dysfunction, demonstrated that all treatment groups, A (control, exercise, and advise), B (exercise, advise, and nonrigid support belt), and C (exercise, advise, and rigid support belt), showed improvement of average pain and significant improvement in function as outcome; however, the difference between groups was not statistically significant, which does not allow for conclusions with regard to the contribution of the belts to the treatment outcomes.

Criteria for methodological quality and completeness of reporting assessment of the studies found were not used in this review for inclusion, due to the limited number of studies available. The current review adheres to PRISMA guideline for systematic reviews.

## 3. Results and Discussion

### 3.1. MSGs Available in the Market

#### 3.1.1. Maternity Support Belts

Yip and Yu [10] defined belts as an elastic panel to be wrapped around the body under the abdomen, frequently recommended for the prevention and treatment of LBP during pregnancy [17]. Support belts are widely commercially available and commonly recommended and used, with strong anecdotal evidence supporting that they are instrumental in reducing fatigue, pressure, stress and strain of the back, preventing and/or relieving back pain, and correcting or improving posture [13, 17, 18].

**Figure 1:**
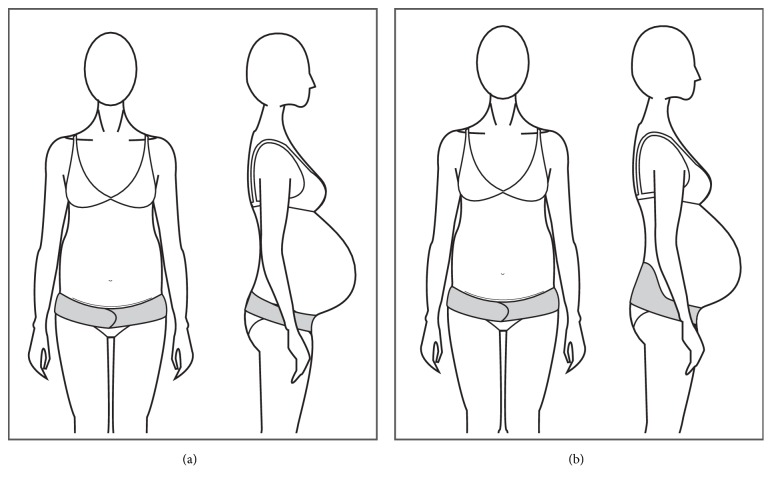
(a) Single panel belt. (b) Wide back belt.

The most common belt designs consist of a one-piece adjustable single panel with or without a wide supporting panel at the back (Figures 1(a) and 1(b)).

Most of the belts available in the market claim to lift the abdomen, support the belly weight, encourage a more erect posture, and relieve pressure off the pelvis, lower back and bladder, offering instant improvement of posture, and reduction and/or elimination of discomforts. Some of the products also claim to decrease varicose veins and hernias and to minimize stretch marks by helping to increase blood flow and circulation.

Most of the belts available mentioned non-evidence-based endorsements from health professionals, as well as anecdotal evidence of effectiveness from previous users of the garments. However, there are only seven belts from different brands which have been scientifically studied previously as sole interventions. They were demonstrated to be effective for improvement of postural instability [19] and reduction in symphyseal pain [20] back pain intensity and duration [11] and/or PGP intensity [21].

Belts come in different sizes, covering women's hips circumferences from 78 cm up to 178 cm, with only one brand (The Ultimate Maternity Belt) offering belts in sizes up to 3XL. The belts range in price from around $20 USD up to $99.95 USD, depending on the width of the belt, its construction and materials, and if the design carries a patent.

Belts have different types of closures and/or extra straps that work as extenders, allowing for growth of the abdominal area. They offer easiness to don and doff, soft fabrics, and possibilities of fitting the garment in different positions, either under the belly or around the hips (high/low) according to individual preference and therapy requirements. Some of the belts offer extra features such as padded backs, pockets for insertion of heat/cold packs, and extra straps that can be fitted around the belly to help with its weight.

Belts are usually made up of synthetic fibres such as nylon, polyester, and elastane and latex free, with advantageous properties such as being lightweight, easy to clean, dimensionally stable, and dirt-resistant [22]; however, the main disadvantage of these fibres is poor moisture absorption which leads to excessive perspiration, elevated body temperature, increased skin sensitivity, and discomfort, affecting the adherence of women to the garment [23, 24].

**Figure 2:**
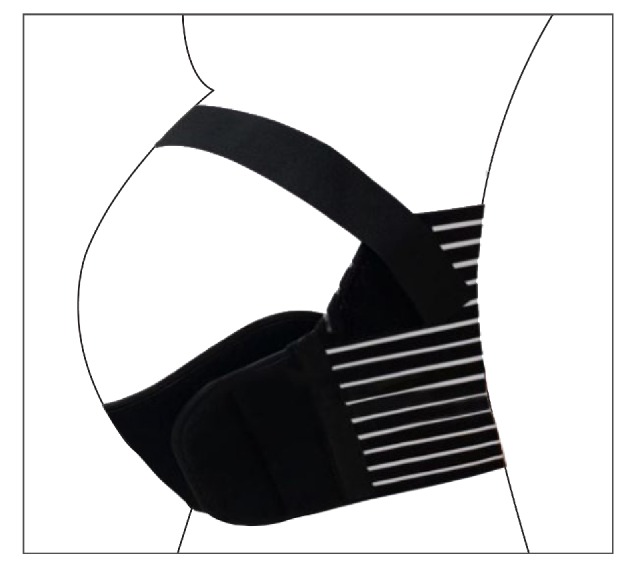
The loving comfort back support.

Carr [11], in a study of the use of belts for LBP alleviation, looked at the acceptability of a wide back belt (Figure 2), concluding that it was effective and rated as very easy to use; however, participants reported discomforts such as skin irritation from the seams and the hook and discomfort in removing the garment to go to the bathroom. It was mentioned that size, weight or obesity did not affect the use of the garment, but height was one of the challenges given the wider back of the belt. The shorter participants complained about feeling the garment back rolled and buckled when sitting.

**Figure 3:**
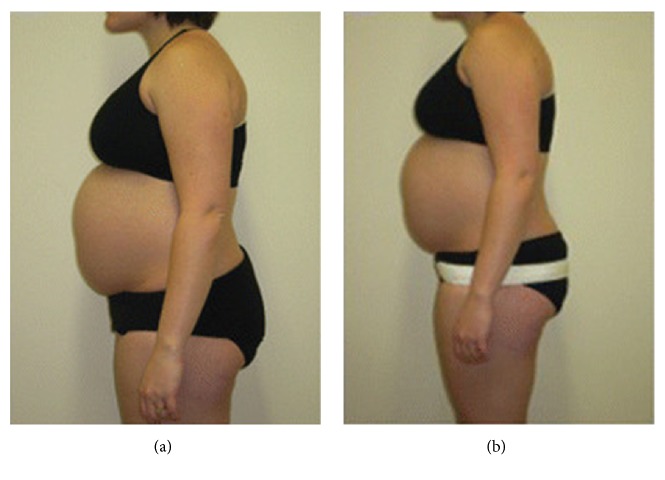
(a) Nonrigid belt (www.smileybelt.co.nz, Havelock North, New Zealand). (b) Rigid belt (The Orthotic Center, Greenlane, Auckland, New Zealand). Source: Flack, Hay-Smith [20]. Image by: Flack, Hay-Smith [20].

Flack, Hay-Smith [20] studied two different pelvic belts (Figures 3(a) and 3(b)) for alleviation of pregnancy-related symphyseal pain and mentioned that the nonrigid belt was regarded as the most effective and comfortable by 82% of the participants, while the rigid belt tended to “ride up” and move out of position when sitting down and was uncomfortable and “digging in” while sitting.

**Figure 4:**
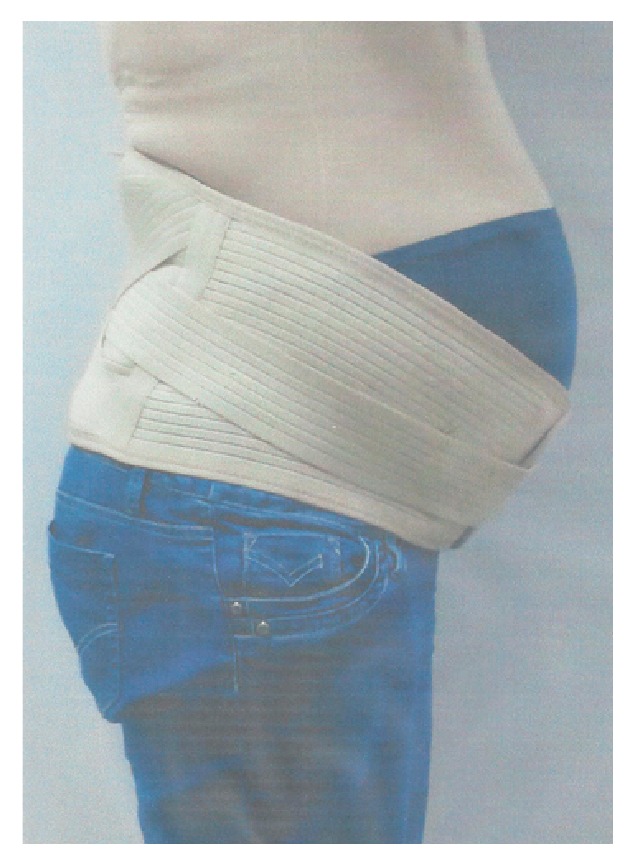
Variteks Ortopedi Sanayi belt Source: Cakmak, Inanir [19] Image by: Cakmak, Inanir [19].

Cakmak, Inanir [19] studied the effect of a nonrigid, elastic belt (Figure 4) on postural balance and concluded that elastic belts are effective to reduce the risk of falling and important for pregnant women and clinicians because of their wearability and adjustability to accommodate the abdominal girth growth.

**Figure 5:**
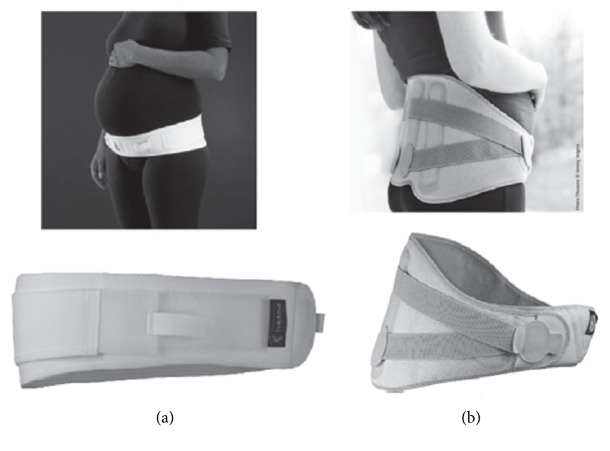
(a) Ortel-P® pelvic maternity belt. (b) LombaMum Maternity Lumbar Brace. Source: Bertuit, Van Lint [25]. Image by: www.thuasne.com.

Finally, the study by Bertuit, Van Lint [25] used two pelvic belts for pregnant women (Figures 5(a) and 5(b)): an adjustable, narrow, and nonrigid belt that can be used in high position at the anterior superior iliac spine or low position at the pubis, and an adjustable wide and rigid belt with metal reinforcements in the lumbar area. The study concluded that both belts helped in global pain intensity, pain intensity at the SIJ, and spine pain.

Several other studies have reported desirable functions of maternity belts: Vleeming, Buyruk [26] found that pelvic belts significantly decreased the sagittal rotation in the SIJ, while Damen, Spoor [27] and Mens, Hoek van Dijke [28] found that wearing a belt below the anterior superior iliac spine significantly reduced SIJ laxity as compared to wearing it at the symphysis level.

#### 3.1.2. Maternity Support Bands

**Figure 6:**
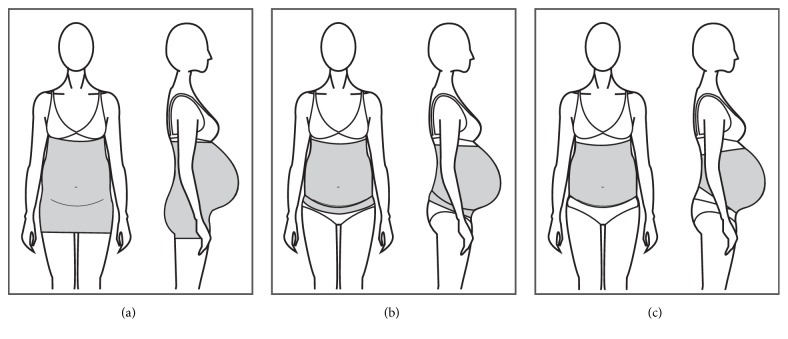
(a) A band covering pelvic area. (b) A band with abdominal support. (c) Short band.

The terms band and belt are sometimes used indistinctively within the products found in the market; however, there is a tendency for defining bands as an elastic, one-piece tubular structure, mostly seamless in construction. The bands are designed to sit under the bust, commonly offering a panel of firm textile material that sits under the belly and an elastic and soft material that sits over the belly to allow the abdomen to grow (Figures 6(a), 6(b), and 6(c)).

**Figure 7:**
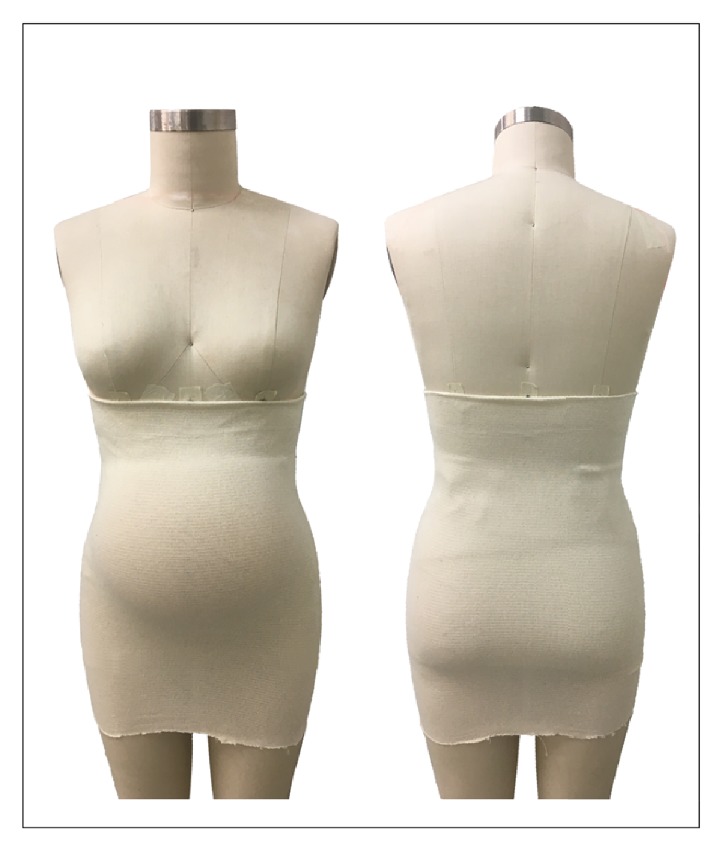
“Tubigrip”.

Only one study was found that investigated the effectiveness of a band on reducing the severity of LBP [12]. The study used a support band as a control garment (Figure 7) and mention the possible mechanical actions of the band as follows: to elevate the weight of the uterus from the symphysis pubis and to compress the pelvic area for stabilization and reduction of pelvic joint motion, concluding that the garment was effective for reducing the severity of LBP and posterior pain in women.

Bands come in different sizes from S to XXL for a hip circumference of up to 115 cm and range in price from around $10 USD up to $60 USD depending on the width of the band and its claimed benefits. They are made of polyester or polyamide and elastomer fibres and are latex free, offering soft touch and reduction of skin irritation.

Most bands claim to provide relief from pregnancy-related back and pelvic pain through the built-in abdominal panel that lifts the abdomen and to reduce the risk of abdominal muscle strains through its compressive effect. Other claims include providing warmth to ease muscles, to provide a smooth look to the belly area, to protect from everyday radiation by the use of specific materials, and to offer antiseptic and antibacterial properties through the use of silver fibres in its construction. There is only one patent found for this type of garments and there is no scientific evidence supporting the claims made by manufacturers.

#### 3.1.3. Full Torso MSGs

**Figure 8:**
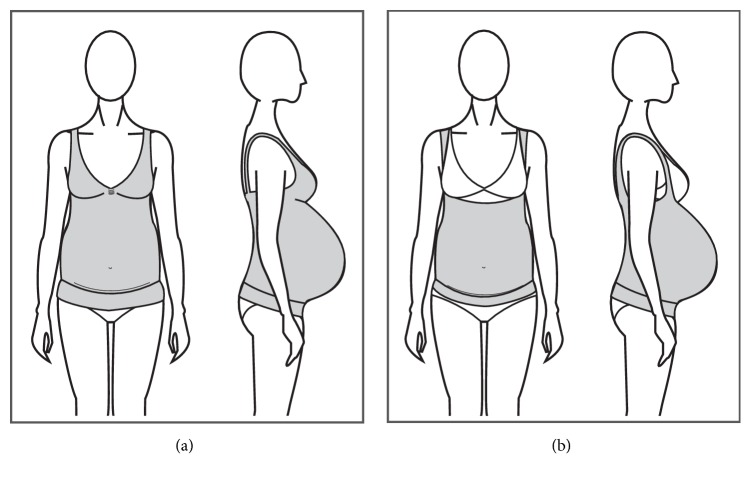
(a) Garment with breast support. (b) Garment without breast support.

Full torso MSGs or maternity vests [29] are one-piece garments that sit on the body as an outerwear top, commonly covering the bust area up to under the belly or under the hips height, with a panel of fabric covering the belly area and an abdominal support panel that sits under the abdomen (Figures 8(a) and 8(b)). Full torso garments offer support to the back and breast areas, helping to alleviate discomforts derived from increased localized weight.

Ho, Yu [22] in a study of comfort evaluation of MSGs, mentioned that full torso garments, compared to garments in the form of briefs, girdles, and belts, were perceived to allow easy movement, convenient for toileting and good to use as underwear during pregnancy but uncomfortable to pull over the shoulders. In another study, Ho [29] considered full torso garments “to be effective in transferring the abdominal weight to other parts of the body (e.g. the shoulders or upper back) when compared to the belt or briefs designs”.

**Figure 9:**
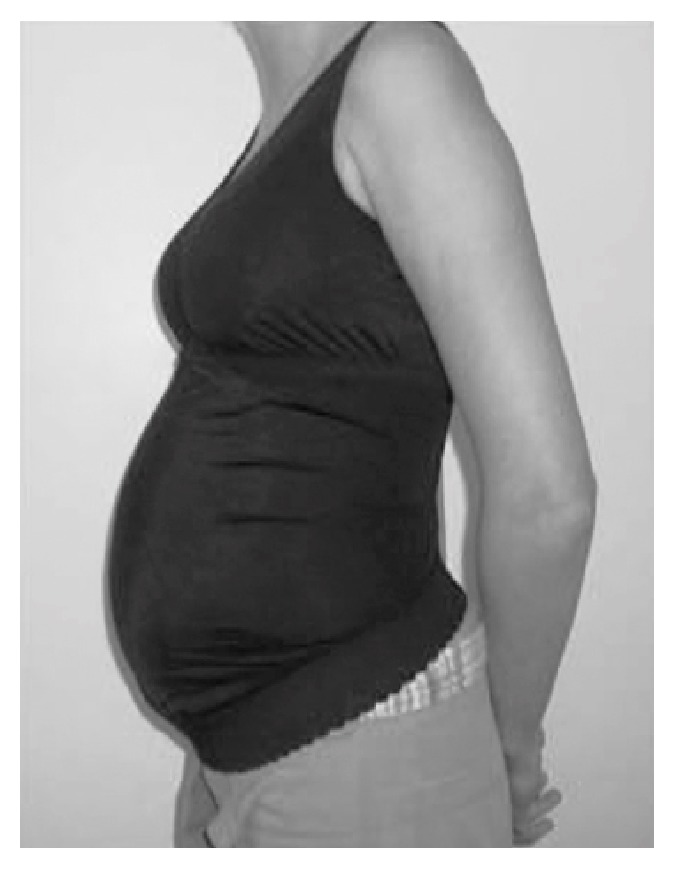
BellyBra® Source: Kalus, Kornman [12] Image by: Kalus, Kornman [12].

In the work of Kalus, Kornman [12], a polyamide/elastane full torso garment was used as the intervention device (Figure 9). It was described as a garment that helped to improve posture through the garment's straps and to support the abdomen and to release weight of the pelvic area through a built-in elastic band that sits under the abdominal area. The results showed that the intervention group had significant reduction of the severity of LBP and posterior pain and its impact on ADL, with no significant change in satisfaction of life scores of participants.

The full torso garments available on the market come in sizes from XS to 2XL and prices ranging from $25 USD up to $75 USD, with most of the garments made from polyamide and elastane fibres and latex free, seamless in construction, and offering moisture wicking and breathable properties. Manufacturers claim to offer back and breast support, improvement of posture, support for the growing belly, reduction of pelvic pressure, and prevention of lumbar pains; most of this is based on anecdotal evidence from health specialists and users.

#### 3.1.4. Maternity Support Cradles

**Figure 10:**
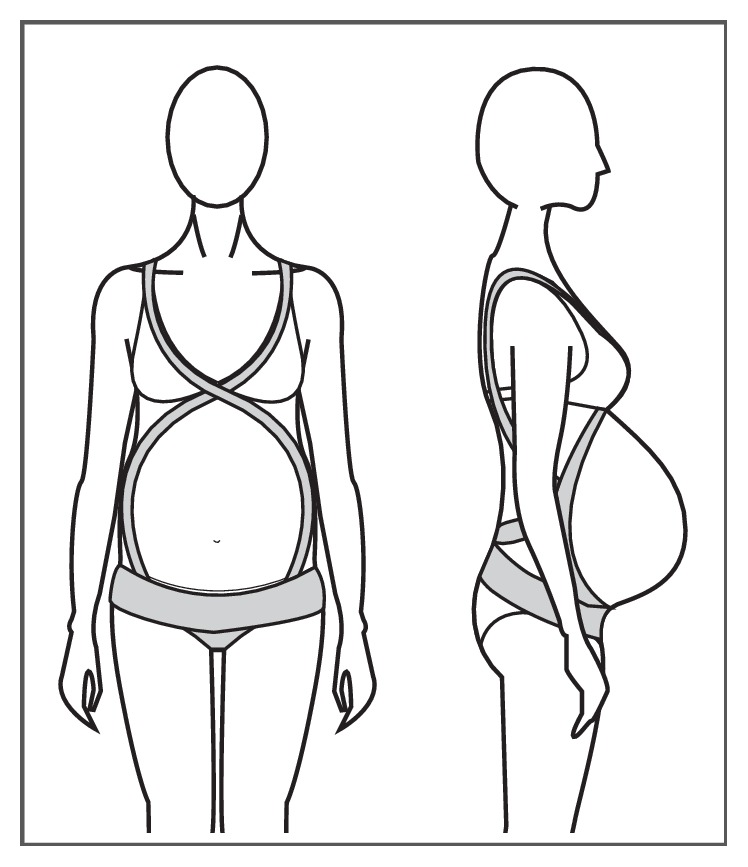
Cradle.

A pregnancy cradle is commonly made of straps of different widths worn across the torso and over the shoulders [10] (Figure 10), made of elastic or non-elastic materials with adjustable straps.

The cradles are available in the market ranging in size from XS to XXL and in prices from $50 USD to $100 USD claiming to offer back pain alleviation, abdominal support, improvement of posture, and pressure relief off the bladder through the use of straps around the shoulders and abdominal areas. Some of the cradles also offer extra straps for support of the pelvic area, claiming to reduce the swelling of veins and the appearance of hernias in the groin area.

Although multiple patents are registered under this type of garment, no scientific studies were found using cradles as the sole intervention, for which its effectiveness is difficult to ascertain.

#### 3.1.5. Maternity Support Brief, Shorts and Legging

**Figure 11:**
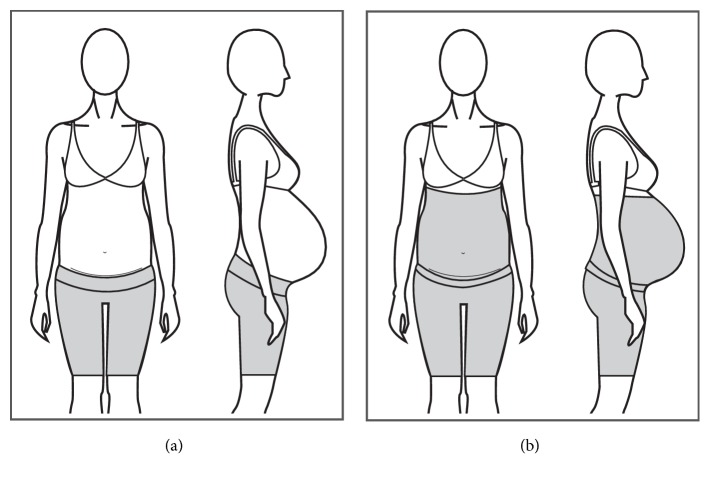
(a) Short with abdominal support. (b) Short with belly coverage.

Briefs, shorts, and leggings are garments commonly made of elastic materials that provide compressive properties to the garment to increase back and pelvic stability of pregnant women (Figures 11(a) and 11(b)). Some garments come with a pouch for the belly to sit in and a built-in panel that sits under the abdomen to provide support to the area.

Yip and Yu [10] mentioned that these types of garments are difficult to put on and take off but can provide well-distributed pressure to the abdominal region, although excessive pressure could cause discomfort and affect blood circulation.

Most of the products in the market claim to reduce back and pelvic pain, to improve pelvic and lumbar stability, to reduce the appearance of varicose veins, to reduce symptoms of incontinence, and to stimulate blood circulation through the compressive effect of the garments. Some of the products also claim to increase mobility and stability of pregnant women and reduction of fatigue and aesthetic benefits such as smooth silhouettes and cellulite control. All of these claims are based on anecdotal endorsements from health professionals and users, with no scientific evidence of their effectiveness as sole intervention.

The garments come in different sizes covering circumferences of up to 163 cm, based on women's measurement of the hips circumference. The length of the garment varies from brief length up to full-leg length and the garments range in price from $40 USD up to $200 USD depending on the garment's length, its construction and materials, and if the design holds a patent.

### 3.2. Effects of MSGs

The literature search generated a wide spectrum of reviews, studies, clinical trials, and reports, of which 24 articles were retrieved. 18 articles were excluded, as they were either review studies or the garments of study were used as part of the treatment and not the sole intervention or were carried out after pregnancy or in nonpregnant women.

In total six peer-review studies were used in this review, of which three are trial studies, two are pilot studies, and one is an observational study. Details of the study design, methods of measurement, and outcome measurements from the selected studies are presented in Table 1. Three outcome measurements were identified from the studies: alleviation of pain, improvement of balance, and improvement of functionality and mobility.

#### 3.2.1. Effect of MSGs on Pain Alleviation

*Low Back Pain*. Although there is a variety of terms used to define LBP during pregnancy and unclear diagnostic criteria [12], multiple studies show that LBP is one of the main discomforts experienced by women, with approximately 50% to 80% of the pregnant population affected [3, 4, 6, 30]. It is reported that LBP affects the performance of ADL during pregnancy [3, 31–33] and is one of the main causes for working women to take sick leave during pregnancy [34, 35].

There are several presentations of pregnancy-related LBP: Carr [11] in her paper mentioned multiple descriptions of LBP such as high back pain, sacroiliac or posterior pelvic pain, and lumbar pain as the most common of them. Albert, Godskesen [36] identified two broad categories of LBP as pain arising from the area of the lumbar spine and pelvic joint pain manifested distal or lateral to the fifth lumbar vertebra. At the same time, there are many causes attributed to LBP such as increase of weight in a specific body part, alterations in posture, muscle fatigue, hormonal changes, and increase of strain in body structures because of the abdominal weight; however, little validation of the hypotheses regarding the causes is available [37].

A variety of trials investigating nonpharmacological treatments for alleviating LBP have been found using treatments such as exercise, progressive muscle relaxation (PRM), spinal manipulative Therapy (SMT), Kinesio Taping (KT), neuroemotional techniques (NET), transcutaneous electrical nerve stimulation (TENS), osteopathic manipulative therapy (OMT), Sham Ultrasound (Sham US), and the use of MSGs.

Only two trials were found to use MSGs as a sole intervention treatment using different types of MSGs (belts and full torso garments) and showing positive results for LBP alleviation and improvement of QOL during pregnancy.

Carr's [11] study investigated the reduction in pain scores and the effect of pain in ADL. The study sample was formed by 30 women allocated to the garment group (Figure 2) and 10 women allocated to a nontreatment group, but due to information contamination during the study, a garment was given to the nontreatment group during the second week of the study. The groups were comparable in gestational age, activities levels, and pain scores before intervention. The participants of this study were at least 20 weeks pregnant with self-reported LBP over the previous week and at least a self-reported “medium” level of pain, with no history of preexisting back pain or disc disease. The participants were asked to wear the garment for two weeks during waking hours.

Pre- and postintervention tests were applied to measure the intensity and duration of pain and its impact on ADL during pregnancy through the pain in pregnancy (PIP) profile questionnaire. Also, a set of activity-related questions asking about the amount of twisting, bending, lifting, walking, sitting, and standing plus open-ended questions about the acceptability of the garment were applied. The intervention group had significantly fewer days of pain after the two weeks of intervention: t (26)= 3.48 and p=0.001, and significantly fewer hours of pain: t(26)= 3.56 and p=0.001 [11], while the comparison group did not have any significant changes in the pain variables over the time during which they did not use the garment. Although the study showed that the intervention group achieved higher LBP relief based on PIP scores, it did not mention the possible mechanism of action of the garments which may influence its effectiveness, neither the interface pressure induced by the garment to the underlying body part, garment construction (fabric and garment details), nor the garments' fitting guidelines.

The study concluded that although there is a need for further study of the effects of MSGs on LBP alleviation, the use of MSGs can significantly reduce pain scores and effects of LBP in women's lives and it also concluded that MSGs could offer a safe, low-cost, and accessible comfort measure for a large number of women affected by LBP during pregnancy.

A second trial that demonstrated the effectiveness of MSGs for alleviation of LBP is the randomized trial by Kalus, Kornman [12], which evaluated the impact of wearing a MSG on LBP severity associated with functional impairment and satisfaction with life (SWL). The garments used during the study were a full torso garment with straps to the shoulders, made out of polyamide and elastane and with an elastic panel that sits under the abdomen (Figure 9), and “Tubigrip” (Figure 7), which is an elastic one-piece tubular structure that sits under the breast area and extends under the pelvic area, used as a control garment. [12]. The study mentioned that the correct garment size was given to the participants as well as instructions on how to wear the garments, but they were free to choose the frequency and duration of wear; however, it is not clear how the garment was selected and fitted and how the difference in frequency and length of wear influenced the outcome measurement.

The study analysed results of 94 women who were betweem 20 and 36 weeks pregnant experiencing LBP or posterior pelvic pain (SIJ) based on oral history and the patient's localization of the pain in a visual chart. 46 women were allocated to the garment of study and 48 to the control garment. The groups were comparable in gestational age, activities levels, and pain scores before intervention.

The study was initiated with participants rating the severity of their pain on a VAS scale by measuring its influence in six physical activities (sleeping, getting up from a sitting position, sitting down, walking, and working) through a Likert scale and by an evaluation of life satisfaction using the Satisfaction With Life Scale (SWLS).

The results showed that the intervention group had a significant reduction of the impact of LBP on sleeping (3.4 versus 4.8; p=0.007), getting up from a sitting position (4.2 versus 5.4; p=0.02), and walking (3.3 versus 5.3; p=0.001) but a low overall impact. The results pointed that both garments showed a significant reduction of the severity of LBP and posterior pain on pregnant women based on VAS scores at baseline and follow-up, but there was no significant change in SWLS scores. There was also less use of analgesic medications by the participants wearing the garment of study compared to the control garment, which infers that the study device was more efficient as a treatment than the control garment. However, the true efficacy of the garments is unclear as some of the participants of both groups used the garment in conjunction with other treatments like physiotherapy, acupuncture, massage, yoga, exercise, heat packs, pillows, and bed rest.

The study reported a statistically significant improvement in alleviation of LBP and a reduction of its impact in the performance of ADL by the use of MSGs; however, there are no details of the garments' construction and materials, recommendations on fitting the garments to the specific body part, hours of use, or pressure requirements which may influence the garments' effectiveness.

*Pelvic Girdle Pain*. PGP is a common complaint during pregnancy. It can be classified into pelvic girdle syndrome (pain in both SIJ and the symphysis pubis), double-sized sacroiliac syndrome (with or without radiation in the sciatic area), and one-sided sacroiliac syndrome (with or without radiation) [12]. The peak of incidence of PGP is 24 to 36 weeks of pregnancy [38] and it affects the performance of ADL during pregnancy [39]. Similar to LBP, PGP causes are not totally understood but the laxity of the sacroiliac joints, lack of stabilization of the region, and biomechanical changes have been mentioned as the main causes of PGP.

Nonpharmacological treatments have been evaluated for alleviation of PGP such as education about body ergonomics and proper body gestures [34, 40], the use of core stabilizing exercises [41], and the use of stabilizing lumbopelvic or sacroiliac belts [30, 38, 40, 42]. It is argued that belts provide pressure that reduce joint's mobility in the pelvic girdle area (sacroiliac and symphyseal) and increase its stability, reducing pain. However, only three studies use MSGs as sole intervention for the alleviation of PGP: Kordi, Abolhasani [21], Flack, Hay-Smith [20], and Bertuit, Van Lint [25]; all of them reporting positive results to PGP alleviation.

The trial by Kordi, Abolhasani [21] studied 105 women between 20 and 32 weeks pregnant with pain in the lumbar region, radiating between the gluteal fold and the posterior iliac crest. The pain was diagnosed based on drawing of the localization of the pain and the results to either Patrick's test or posterior pelvic pain provocation test or a modified Trendelenburg test and direct palpitation of symphysis pubis test. Participants were allocated to 3 different groups: information group that received information about anatomy, body posture and ergonomic advice regarding sitting, walking, and lying; belt plus information group that received the same information as previous group plus a nonrigid lumbopelvic belt of undisclosed style, brand, and features to use during waking times; and information plus a home exercise program group that received the same information as group one plus an exercise program designed to strengthen the pelvic girdle muscles. The groups were comparable at baseline and had follow-up visits at weeks three and six of the study.

The pain intensity measurement in this trial used a validated Persian version of the Oswestry Disability Index (ODI) questionnaire, a validated Persian version of World Health Organization's QOL Questionnaire (WHOQOL-BREF) which contains four different categories and aspects of QOL including physical health and psychological health as well as social and environmental conditions, and a VAS scale. The study showed a statistically significant decrease in pain scores and ODI scores (p<0.001) by the belt plus information group at three and six weeks of the study compared to the other two groups. It was also shown that the scores of the WHOQOL-BREF questionnaire in the group using the belt were significantly higher than the other two groups in all components but the social category.

This study demonstrated the efficiency of a MSG in the form of a lumbopelvic belt (plus ergonomic information to patients) in alleviation of PGP; however, there is not much information about the type of belt used during the study and the difference between the lumbopelvic and sacroiliac belts mentioned as to understand the potential mechanics of the garments of study and their influence in the therapy's effectiveness.

In a second study, Flack, Hay-Smith [20] focused on the adherence (frequency and duration of use), tolerance (comfort), and effectiveness (symptomatic relief) of two different pelvic belts as sole treatment for pubic symphyseal pain alleviation, which is a distinct subgroup of PGP [43]. The study used a nonrigid belt made of neoprene material (Figure 3(a)) and a rigid belt that is “thinner and made of nylon webbing and lined with foam” (Figure 3(b)), both worn in “low" position. The study involved 20 participants experiencing pubic symphyseal pain for at least two weeks (worse than any concurrent posterior pelvic pain) who responded positively to at least two of the three tests applied: reproduction of pain from palpation, modified Trendelenburg's test, and active straight leg raise test. The study evaluated symphyseal pain intensity, influence on ADL, influence on disability, and joint hypermobility.

The pain intensity assessment was done through a VAS scale, the influence of symphyseal pain on ADL was determined by the Modified Oswestry Disability Questionnaire (MODQ), the joint hypermobility was measured using a modified nine-point Beighton Hypermobility Score, and the Patient Specific Functional Scale (PSFS) was completed to evaluate the influence of pain on disability. All scores were measured at baseline and at the end of week three of study.

Women were randomized to either a nonrigid or rigid belt in comparable groups at baseline, advised on how to use the belt by a physiotherapist and asked to wear it during wake hours. Although the study mentioned that the participants were shown how to use the belts in a low position (over the pubic symphysis), the study does not describe the required compression of the garment to the body part. The participants were interviewed weekly over the phone as to complete the PSFS and determine adherence and tolerance of the belt. At the same time, participants responded to a daily text message that asked about changes in pain intensity and changes in ability to perform functional activities. After three weeks, the participants were fitted with the alternate belt to wear for one week following the same methodology as with the initially allocated belt.

The results of this study showed a reduction in PSFS scores by 36% in the nonrigid belt group and 34% in the rigid belt group. It showed that rolling over in bed, walking, and getting up from sitting were the activities particularly difficult to perform by women but improved with the use of MSGs. VAS scores were also significantly decreased in both groups (p=0.018) but there was not a significant change in overall MODQ or PSFS scores. Nonrigid belts showed a higher reduction of scores than the rigid belt and were the preferred MSG in terms of comfort. However, the study did not mention the potential mechanisms that influence the garments' effectiveness on alleviating pain and discomfort, neither was there an objective analysis of the comfort properties of the garment and its comprising materials.

Pelvic belts were worn an average of 4.9 ± 2.9 hours per day with no significant difference in hours of wear between groups. Longer periods of use were associated with a greater decrease in VAS scores but not different in PSFS (p=0.546) or MODQ (p=0.096) scores. Although the results are positive for the use of MSGs for alleviation of PGP and improvement in functional mobility, the study does not show a well-defined methodology for garment application (fit, compressive requirements and hours of use) which may influence its effectiveness; also, the sample size was small to generalize results, so there is a need for a larger study to confirm the initial findings.

Finally, the trial by Bertuit, Van Lint [25] focused on the study of the effectiveness of support belts as a sole intervention for alleviation of PGP and improvement of the functional capacity of women during pregnancy. The study used two belts: a narrow and nonrigid belt (Figure 5(a)) and an adjustable wide and rigid belt with metal reinforcements in the lumbar area (Figure 5(b)); however, how the garments were fitted and adjusted to the body and the compression provided to the wearer were not discussed in the study.

The study involved 46 pregnant women from the 18th week of pregnancy, experiencing pain in the SIJs and/or pubic region, who responded positively to at least two of the following tests: posterior pelvic pain provocation test, Patrick Faber's test, Trendelenburg modified test, pain provocation tests, and active straight leg raise test, with 59% of participants presenting concurrent LBP. PGP was experienced by women as deep (63%), diffuse (56%), and irradiating pain (34%) and located at the gluteal region (43%), the iliac crest (43%), the groin (19%), and the pubic area (17%) [25].

The quantitative evaluation of pain was done through the VAS and a qualitative assessment through a topographic representation. The Quebec Back Pain Disability Scale (QBPDS) was used to assess the functional capacity of women to develop activities such as sitting, walking, and prolonged standing. All measurements were done at the start of the study (T1) and at the 34th week of pregnancy (T2). Women were randomized into two comparable groups: group A that wore a belt and group B that did not. Group A was also randomized into two groups: group of narrow and nonrigid belt and group of wide and rigid belt.

There is no information on how the participants were advised to use the belts (hours and position), but the frequency of use was reported as several times a week by 68% of participants (average of four days a week and 2 hr 30 min/day). The results of this study showed that women wore the belts for daily activities (55%), going out (42%), and gait (37%), with 48% of women reporting decrease in pain and 63% of women reporting feeling increased support. The study reported no significant differences in pain reduction between the two different belts of study; however, the narrow belts group showed a significant decrease in global pain intensity (p<0.001) and pain intensity at the SIJ (p=0.003), while the wider belts group showed a significant decrease of spine pain (p=0.01).

All studies reported reduction of PGP with the use of MSGs; however, the information about the garments' application method, garments' materials and construction, the interface pressure induced by the garments to the underlying body part, garments' pressure distribution, and possible mechanics of action of the garments remains scarce in all studies reviewed.

#### 3.2.2. Effect of MSGs on Balance

During pregnancy, women are at higher risk of falls due to the anatomical, hormonal, and physiological changes that happen during gestation [19]. Weight gain, increase in joint laxity, increase of spinal lordosis, decrease of neuromuscular control and coordination, and changes in biomechanics and the centre of body mass have been mentioned as the main changes that affect balance of women during pregnancy [41, 44, 45]. 27% of pregnant women fall during pregnancy with 10% of them experiencing two or more falls [46] resulting in injuries such as bone fractures, joint sprains, muscle strains, head injury, rupture of internal organs, internal haemorrhage, abrupt placenta, rupture of the uterus and membranes, and occasionally maternal death or intrauterine foetal demise [47, 48].

The study by Cakmak, Inanir [19] investigated the effect of a support belt (Figure 4) on postural stability during pregnancy. The study recruited 90 women aged 18 to 40 years in any trimester of pregnancy estimated by the date of confinement. The participants were divided into three groups, based on the trimester of gestation. The groups were comparable in age, gravity, parity, and height but not weight and body mass index, given the differences between trimesters. The size of the belt allocated to each participant was defined based on the height and weight of the participants and fitted at the level of the anterior superior iliac spine in the lateral sides, the lower lumbar region around the back, and between the pubis and the umbilicus in the front. Although the belts of study were available in small (70 cm length and 15x25 cm anterior-posterior width), medium (90 cm length and 20x30 cm anterior-posterior width), and large (100 cm length and 25x35 cm anterior-posterior width) sizes, the garments' selection process to fit the different height and weights of the participants is not clear, which may have an impact in the therapy effectiveness.

The dynamic postural stability test was performed to evaluate the postural stability of women, by using the Biodex Balance System (BBS version 3.1) which consists of a movable balance platform that provides up to 20 degrees of surface tilt in a 360-degree range of motion. The measurement includes the overall stability index score (OA), the anterior-posterior stability index (APSI), the medial-lateral stability index (MLSI), and the risk of falling test score (FRT). The range of scores was between 0 degrees and 20 degrees in all stability indexes, with high scores indicating a poor balance. All measurements were obtained by mean of three times and 20 seconds' intervals. Four measurements from the BBS were compared between pregnant women with and without a belt.

The study demonstrated an increase of OA, APSI, MLSI, and FRT scores when comparing the three groups from the first trimester to the third trimester. The FRT scores were significantly lower in each trimester with the use of the belt but higher in the third trimester compared to the other two trimesters. The mean score of FRT was 0.99 ± 0.26 in the first-trimester group and SD= 1.27 ± 0.52 and 1.72 ± 1.03 (p=<0.001) for the second- and third-trimester groups, confirming the decrease in the risk of falling for each group. These results confirmed the positive effects of MSGs in improving impaired balance and reducing the risk of falling in any trimester of pregnancy.

Although the study showed that MSGs does not limit the range of motion of pregnant women during any trimester which is important for therapy adherence, the effects of the garment in a longer period of time are not clear as well as the mechanical action of the garments to improve postural stability. Only one study was found to investigate the effect of MSGs on balance during pregnancy, which shows a need for further studies to confirm the findings.

#### 3.2.3. Effect of MSGs on Functionality and Mobility

Multiple studies have reported that approximately 50%-80% of women experience pregnancy-related pains and discomforts [3, 4, 30, 32, 49] with symptoms varying in duration and intensity and compromising the performance of ADL such as sleeping, sitting, walking up the stairs, bending, and general walking [3, 31, 32, 50], impacting QOL during pregnancy.

Several authors agree that physical discomforts may be disabling conditions during pregnancy and are associated with the changes in body shape, weight increase, and hormonal and musculoskeletal changes. However, as the discomforts are transient and considered as expected or normal during pregnancy, this area of study has become under researched. Although there are no specific studies investigating the impact of MSGs as a sole intervention for improvement of functionality and mobility during pregnancy, majority of studies in this review reported the effect of the garments in functional mobility as a secondary outcome.

Carr's [11] study showed that, based on the scores collected in the PIP questionnaire, the intervention group was less affected for activities such as house/yard work, t(26)=3.24, p=0.001; family activities, t(26)=2.98, p=0.01; and exercise, t(26)=3.63, p=0.001. The study by Kordi, Abolhasani [21] showed an improvement of the functional status of the participants in the group wearing the garment and higher scores in WHOQOL-BREF questionnaire.

The study by Flack, Hay-Smith [20] showed that the activities that women had difficulty with were rolling over in bed (n= 11/20), walking (for a variable time, n= 8/20), and getting up from sitting (after a variable period spent sitting, n=7/20), with MSGs improving women's functionality based on PSFS scores, although there was no improvement of MODQ scores. The study by Kalus, Kornman [12] also concluded that the garments were effective in reducing the impact of pain in physical activities such as sleeping, getting up from a sitting position, walking, and working; and although the garments were recommended for wear during waking and/or active hours, a positive impact of its use in the sleeping activity was reported with no further suggestions on how the garment's mechanism of action might influence the effects after wear.

Finally, the study by Bertuit, Van Lint [25] also evaluated the effects of MSGs on the functional capacity of women during pregnancy showing that the activities that increased or caused PGP were determined as prolonged standing (58%) or sitting (52%), walking (56%), and all activities (50%), with a QBPDS mean score of 41/100, determining that women had significant disabilities for the performance of ADL, which increased with the pass of the day or amount of activities; however, 15% of participants experienced pain continuously, chronic and latent even without performing any activity. The study concluded that pain of participants decreased with the longer use of the belts of study, improving the performance of ADL.

Although the results of the researches reviewed in this study showed positive outcomes in the improvement of functionality and mobility by the use of MSGs, the evidence is not conclusive and raises the need for further objective studies that quantify the comfort improvement of pregnant women by the use of MSGs.

### 3.3. Suggested Mechanism of Action of MSGs

The studies reviewed have shown positive results for the use of MSGs for LBP, PGP, and symphyseal pelvic pain alleviation, as well as improvement of functional mobility of pregnant women and reduction of risk of falling during pregnancy; however, only a few studies hypothesised over the potential mechanisms of action of the garments.

Carr [11] suggested that the belt used during the study provides “low back support (lumbosacral area), stabilizes the pelvis, and elevates some of the weight of the uterus from the symphysis pubis by supporting the lower abdomen”; however, these aspects are not demonstrated or quantified by the study.

Flack, Hay-Smith [20] also mentioned that the mechanism of the belts used in their study was uncertain but suggested that improved proprioception may be influencing the behaviour of participants as having an external apparatus (belt) will make pregnant women aware of the activities undertaken by the body and will help women to modify behaviour to reduce pain.

Bertuit, Van Lint [25] suggested that the use of pelvic belts may produce two different effects on pregnant women: a proprioceptive effect that improves body perception and movement awareness of the wearer and a second effect in which the garment acts as a modulating system to block pain influx conveyed at the spinal level. The study also suggested that belts could have a biomechanical effect to relieve and stabilise the SIJ; however, this was not an objective of the study and was not characterized.

Finally, Kalus, Kornman [12] suggested that the garment of study that had shoulder straps helped improving women's posture and that the garment was designed to provide support through an “elastic band that sits below the abdomen supporting the uterus and lifting the weight off the pelvis”; however, no further details were provided on the garments' mechanisms of action to provide these benefits to the wearer. Also, as the intervention and control garments were effective in reducing LBP, the study mentioned that it is not known if the efficacy of the garments relies on a true efficacy or a placebo effect.

All other studies that are part of this review did not include any information regarding the mechanics of action of the MSGs of study, making this a necessary area of future research.

## 4. Conclusions

This review paper is, to our knowledge, the first to formally review the use of MSGs as sole therapeutic intervention for alleviation of pregnancy-related pains and discomforts, revealing that wearing MSGs during pregnancy could have beneficial effects when worn by pregnant women such as pain reduction (LBP, PGP, posterior pelvic, pubic symphyseal, and pain in the SIJ), improvement of functionality and mobility, and reduction of risk of fall during pregnancy; however, the mechanisms of the garments' actions, as well as the duration of the garments' effectiveness are not elucidated through the studies. Only six studies were found to investigate MSGs as sole intervention for pain and discomfort alleviation during pregnancy, limiting the conclusions of this review.

There are multiple types of MSGs commercially available, with manufacturers claiming to provide different benefits and comfort improvement to the wearer through the design, materials, and/or constructions of their garments; however, none of them provide scientific evidence to support their claims. This review also showed that there is a wide variety of garments used in the studies with the belts being the most popular across them for alleviation of LBP and PGP; nonetheless, it is unclear why the selection of the specific garment for the studies was made. Only the study by Kalus, Kornman [12] mentioned that the control garment used during the study was selected because it was a generic form of support and commonly used at several Australian hospital sites. Also, across the studies reviewed, it was recommended for the garments to be used during waking and or/activity time with no clear number of hours required for the garment to be worn, and no clear methods of selection and application (size, fit, pressure, and type of garment) which may influence the garments' effectiveness.

Although the evidence is strong in showing the garments' effectiveness in pain alleviation, improvement of balance, and improvement of functionality and mobility of pregnant women, the mechanism of action of the garment on the body is not clear for it to provide benefits to the wearer. It was proposed that either a stabilization of the pelvic area or increase in body proprioception were the actions of the garments on the pregnant women. Also, the studies did not provide information regarding the garment's materials and construction and its influence on comfort of women during pregnancy, nor an understanding of the interface pressure induced by the garments to the different body parts or its distribution in the body area and the optimal pressure required by the wearer for the specific therapeutic application.

It will be important not only to establish the clinical recommendations for the selection and application of the garment to the specific therapeutic use and wearer's needs, but also to evaluate the construction and physical properties of the fabrics used in the garments to understand their impact on garment's effectiveness and the wearer's comfort as well as the possible mechanical effects of the garments on the underlying body part.

## Figures and Tables

**Table 1:** Summary of studies on the effectiveness of MSGs.

References	Study Design	Sample size	Gestational Age	Type of Pain	Pain verification	Study duration	Garment used	Outcome measures	Method of measurement	Conclusion of the study
Carr [11]	Pilot study, prospective, nonrandomized, two-group design with repeated measures	Intervention group: 30 women Comparison group: 10 women	At least 20 weeks	Low back pain, excluding women with preexisting back pain or disc disease	Self-report LBP over the previous week at least at a “medium” level	2 weeks	(i)Support Belt/Binder: The Loving Comfort lumbosacral orthosis	Low back pain intensity and duration	Pain in pregnancy profile (PIP): scale: 0 “no pain" to 10 “the worst pain"	The use of a support belt for LBP is effective in reducing pain scores and improving PIP scores. The belt of study is easy to use and well accepted by pregnant women.
								Influence of pain on ADL	Activity-related effect of pain on activities: scale 0 “all the time" to 7 “never" plus nonapplicable option (on pain in pregnancy (PIP) questionnaire)	
								Acceptability of the support garment	Open-ended questions about acceptability of the support garment	
Kalus, Kornman [12]	Randomized controlled trial	94 women	20-36 weeks	Lumbar back pain or posterior pelvic (SIJ)	Based on an oral history and on the patient's localization of their pain on a visual back chart	3 weeks	(i) Full torso garment: Belly Bra® (intervention) (ii) “Tubigrip” (control)	Low back and posterior pelvic pain severity	Visual Analogue Scale (VAS)	The garments used during the study are effective for reducing the severity of LBP, with Belly Bra® being more effective in alleviating the impact of pain on specific ADL.
								Influence of pain in physical activity	Likert scale	
								Satisfaction with life	Satisfaction With Life Scale (SWLS)	
Kordi, Abolhasani [21]	Randomized controlled trial, 3 groups of study	96 women	21-30 weeks	Pelvic girdle pain	Pain drawing and positive result of one of the two following tests: (i) Patrick's test and posterior pelvic pain provocation test for patients with more pain around the SIJ (ii) Modifying Trendelenburg test and direct palpation of the symphysis pubis test for patients with more complaints in symphysis pubis	6 weeks	(i) Nonrigid lumbopelvic belt	Pelvic girdle pain intensity	Visual Analogue Scale (VAS)	The combination of the use of a lumbopelvic belt with information about ergonomics and anatomy of the spine during pregnancy is more effective than the combination of exercise and information to reduce PGP and to improve functional mobility of women during pregnancy, improving QOL
								Quality of life	World Health Organization's Quality of Life Questionnaire (WHOQOL-BREF)	
								Functional status	Oswestry Disability Index Questionnaire (ODI)	
Cakmak, Inanir [19]	Prospective and observational cohort study	90 women: 30 per trimester	First, second, and third trimesters	N/A	N/A	Not mentioned	(i) Maternity support belt: Variteks Ortopedi Sanayi	Postural stability	Overall Stability Index (OA) - level 8 - range of scores from O° to 20°	(i) MSGs are useful for improvement of impaired balance and FRT scores across all trimesters of gestation, helping to reduce the risk of falling of pregnant women
									Anterior-posterior stability index (APSI) - level 8 - range of scores from O° to 20°	
									Medial-lateral stability index (MLSI) - level 8 - range of scores from O° to 20°	
									Fall Risk Test (FRT) - level 8 - range of scores from O° to 20°	
Flack, Hay-Smith [20]	Unblinded, single-center, 2-arm, parallel-group randomized pilot trial	20 women	29-38 weeks	Pubic symphyseal pain	A positive response to at least two of three clinical tests: reproduction of pain from palpation, modified Trendelenburg's test, or active straight leg raise test	3 weeks	(i) Rigid belt: LC symphysis pubis belt (The Orthotic Center) (ii) Nonrigid belt: Smiley belt	Symphyseal pain intensity	Visual Analogue Scale (VAS)	(i) Pelvic support belts may have a positive effect in the reduction of pubic symphyseal pain and improvement of functionality in pregnant women. (ii) Nonrigid belts may be more comfortable and effective for managing pelvic symphyseal pain than rigid belts
								Influence of symphyseal pain on ADL	Modified Oswestry Disability Questionnaire (MODQ)	
								Influence of symphyseal pain on disability	Patient Specific Functional Scale (PSFS)	
								Joint hypermobility	Nine-point Beighton Hypermobility Score	
Bertuit, Van Lint [25]	Randomized control trial, two-group longitudinal study	46 women	From 18 weeks	Pain in the SIJ and/or pubic region, excluding women with presence of lumbar-pelvic pain before pregnancy	Positive result for at least half of the following set of tests: posterior pelvic pain provocation test, Patrick Faber's test, Trendelenburg modified test, pain provocation tests, and active straight leg raise test during clinical examination	(i) Measurement 1 at start of study (ii) Measurement 2 at 34 weeks of pregnancy	(i) Ortel-P® Pelvic Maternity Belt-Thuasne belt (ii) Lomba Mum Maternity Lumbar Brace-Thuasne	Pelvic girdle pain	Visual Analogue Scale (VAS)	The use of maternity support belts reduced PGP, particularly on the SIJ over a 9-week period by increasing women proprioception and biomechanical effects. The use of belts improved the performance of ADL by pregnant women.
									Topographic representation	
								Functional capacity	Quebec Back Pain Disability Scale (QBPDS)	


## References

[1] Kristiansson P., Svärdsudd K., Von Schoultz B. (1996). Back pain during pregnancy: a prospective study. *The Spine Journal*.

[2] Liddle S. D., Pennick V. (2015). Interventions for preventing and treating low-back and pelvic pain during pregnancy. *Cochrane Database of Systematic Reviews*.

[3] Wang S.-M., Dezinno P., Maranets I., Berman M. R., Caldwell-Andrews A. A., Kain Z. N. (2004). Low back pain during pregnancy: prevalence, risk factors, and outcomes. *Obstetrics & Gynecology*.

[4] Mogren I. M., Pohjanen A. I. (2005). Low back pain and pelvic pain during pregnancy. *The Spine Journal*.

[5] Fitzgerald C. M., Segal N. A., Fitzgerald C. M. e., Segal N. A. e. (2015). *Musculoskeletal Health in Pregnancy and Postpartum: An Evidence-Based Guide for Clinicians*.

[6] Wu W. H., Meijer O. G., Uegaki K. (2004). Pregnancy-related pelvic girdle pain (PPP), I: terminology, clinical presentation, and prevalence. *European Spine Journal*.

[7] Vleeming A., Albert H. B., Östgaard H. C., Sturesson B., Stuge B. (2008). European guidelines for the diagnosis and treatment of pelvic girdle pain. *European Spine Journal*.

[8] Sinclair M., Close C., McCullough J. E., Hughes C., Liddle S. D. (2014). How do women manage pregnancy-related low back and/or pelvic pain? Descriptive findings from an online survey. *Evidence Based Midwifery*.

[9] Vermani E., Mittal R., Weeks A. (2010). Pelvic girdle pain and low back pain in pregnancy: a review. *Pain Practice*.

[10] Yip J., Yu W. (2006). *Innovation and Technology of Women's Intimate Apparel*.

[11] Carr C. A. (2003). Use of a maternity support binder for relief of pregnancy-related back pain. *Journal of Obstetric, Gynecologic, & Neonatal Nursing*.

[12] Kalus S. M., Kornman L. H., Quinlivan J. A. (2008). Managing back pain in pregnancy using a support garment: a randomised trial. *BJOG: An International Journal of Obstetrics & Gynaecology*.

[13] Ho S., Luo Y., Yu W., Chung J. (2006). Physical and physiological health effects of intimate apparel. *Innovation and Technology of Women's Intimate Apparel*.

[14] Johnson L. M. (2011). Effective manual therapy intervention for pubic symphyseal pain in a postpartum patient. *Journal of Womens Health Physical Therapy*.

[15] Mens J. M., Vleeming A., Snijders C. J., Stam H. J., Ginai A. Z. (1999). The active straight leg raising test and mobility of the pelvic joints. *European Spine Journal*.

[16] Depledge J., McNair P. J., Keal-Smith C., Williams M. (2005). Management of symphysis pubis dysfunction during pregnancy using exercise and pelvic support belts. *Physical Therapy in Sport*.

[17] Ho S. S., Yu W. W., Lao T. T., Chow D. H., Chung J. W., Li Y. (2009). Effectiveness of maternity support belts in reducing low back pain during pregnancy: a review. *Journal of Clinical Nursing*.

[18] Yu W., Wong W. Design and development of maternity supportive undergarment.

[19] Cakmak B., Inanir A., Nacar M. C., Filiz B. (2014). The effect of maternity support belts on postural balance in pregnancy. *PM&R : The Journal of Injury, Function, and Rehabilitation*.

[20] Flack N. A. M. S., Hay-Smith E. J. C., Stringer M. D., Gray A. R., Woodley S. J. (2015). Adherence, tolerance and effectiveness of two different pelvic support belts as a treatment for pregnancy-related symphyseal pain - a pilot randomized trial. *BMC Pregnancy and Childbirth*.

[21] Kordi R., Abolhasani M., Rostami M., Hantoushzadeh S., Mansournia M. A., Vasheghani-Farahani F. (2013). Comparison between the effect of lumbopelvic belt and home based pelvic stabilizing exercise on pregnant women with pelvic girdle pain; a randomized controlled trial. *Journal of Back and Musculoskeletal Rehabilitation*.

[22] Ho S. S., Yu W., Lao T. T., Chow D. H., Chung J. W., Li Y. (2008). Comfort evaluation of maternity support garments in a wear trial. *Ergonomics*.

[23] Cherry S. H., Moss D. G. (2004). *Understanding Pregnancy and Childbirth*.

[24] Johnson J., Greenspan B., Gorga D., Nagler W., Goodwin C. (1994). Compliance with pressure garment use in burn rehabilitation. *Journal of Burn Care & Rehabilitation*.

[25] Bertuit J., Van Lint C. E., Rooze M., Feipel V. (2018). Pregnancy and pelvic girdle pain: Analysis of pelvic belt on pain. *Journal of Clinical Nursing*.

[26] Vleeming A., Buyruk H. M., Stoeckart R., Karamursel S., Snijders C. J. (1992). An integrated therapy for peripartum pelvic instability: a study of the biomechanical effects of pelvic belts. *American Journal of Obstetrics & Gynecology*.

[27] Damen L., Spoor C. W., Snijders C. J., Stam H. J. (2002). Does a pelvic belt influence sacroiliac joint laxity?. *Clinical Biomechanics*.

[28] Mens J., Hoek van Dijke G., Pool-Goudzwaard A., van der Hulst V., Stam H. (2006). Possible harmful effects of high intra-abdominal pressure on the pelvic girdle. *Journal of Biomechanics*.

[29] Ho S-mS. (2008). *Maternity Garment Treatment for the Relief of Low Back Pain*.

[30] Berg G., Hammar M., Möller-Nielsen J., Lindén U., Thorblad J. (1988). Low back pain during pregnancy. *Obstetrics & Gynecology*.

[31] Hansen A., Jensen D. V., Wormslev M. (2000). Pregnancy associated pelvic pain. II: Symptoms and clinical findings. *Ugeskr Laeger*.

[32] Nazik E., Eryilmaz G. (2013). Incidence of pregnancy-related discomforts and management approaches to relieve them among pregnant women. *Journal of Clinical Nursing*.

[33] Shinkawa H., Shimada M., Hirokane K., Hayase M., Inui T. (2012). Development of a scale for pregnancy-related discomforts. *Journal of Obstetrics and Gynaecology Research*.

[34] Noren L. R. P. T., Östgaard S., Nielsen T. F., Östgaard H. C. (1997). Reduction of sick leave for lumbar back and posterior pelvic pain in pregnancy. *The Spine Journal*.

[35] Ostgaard H. C. (1996). Assessment and treatment of low back pain in working pregnant women. *Seminars in Perinatology*.

[36] Albert H. B., Godskesen M., Westergaard J. G. (2002). Incidence of four syndromes of pregnancy-related pelvic joint pain. *The Spine Journal*.

[37] Perkins J., Hammer R. L., Loubert P. V. (1998). Identification and management of pregnancy-related low back pain. *Journal of Nurse-Midwifery*.

[38] Ostgaard H. C., Zetherstrom G., Roos-Hansson E., Svanberg B. (1994). Reduction of back and posterior pelvic pain in pregnancy. *The Spine Journal*.

[39] Elden H., Ostgaard H.-C., Fagevik-Olsen M., Ladfors L., Hagberg H. (2008). Treatments of pelvic girdle pain in pregnant women: adverse effects of standard treatment, acupuncture and stabilising exercises on the pregnancy, mother, delivery and the fetus/neonate. *BMC Complementary and Alternative Medicine*.

[40] Nilsson-Wikmar L., Holm K., Öijerstedt R., Harms-Ringdahl K. (2005). Effect of three different physical therapy treatments on pain and activity in pregnant women with pelvic girdle pain: a randomized clinical trial with 3, 6, and 12 months follow-up postpartum. *The Spine Journal*.

[41] Dumas G., Reid J., Wolfe L., Griffin M., McGrath M. (1995). Exercise, posture, and back pain during pregnancy. *Clinical Biomechanics*.

[42] Mens J. M., Vleeming A., Stoeckart R., Stam H. J., Snijders C. J. (1996). Understanding peripartum pelvic pain: implications of a patient survey. *The Spine Journal*.

[43] Albert H., Godskesen M., Westergaard J. (2001). Prognosis in four syndromes of pregnancy-related pelvic pain. *Acta Obstetricia et Gynecologica Scandinavica*.

[44] Calguneri M., Bird H. A., Wright V. (1982). Changes in joint laxity occurring during pregnancy. *Annals of the Rheumatic Diseases*.

[45] McCrory J., Chambers A., Daftary A., Redfern M. (2010). Dynamic postural stability during advancing pregnancy. *Journal of Biomechanics*.

[46] Dunning K., Lemasters G., Levin L., Bhattacharya A., Alterman T., Lordo K. (2003). Falls in workers during pregnancy: risk factors, job hazards, and high risk occupations. *American Journal of Industrial Medicine*.

[47] Mirza F., Devine P., Gaddipati S. (2010). Trauma in pregnancy: a systematic approach. *American Journal of Perinatology*.

[48] Fildes J., Reed L., Jones N., Martin M., Barrett J. (1992). Trauma: the leading cause of maternal death. *Journal of Trauma and Acute Care Surgery*.

[49] Fox P., Yamaguchi C. (1997). Body image change in pregnancy: a comparison of normal weight and overweight primigravidas. *Women and Birth*.

[50] Shinkawa H., Shimada M., Hirokane K., Hayase M., Inui T. (2012). Development of a scale for pregnancy-related discomforts. *Journal of Obstetrics and Gynaecology Research*.

